# Endothelial Agrin Is Dispensable for Normal and Tumor Angiogenesis

**DOI:** 10.3389/fcvm.2021.810477

**Published:** 2022-01-31

**Authors:** Peng Ye, Zelong Fu, Jeff Yat-Fai Chung, Xiaoyun Cao, Ho Ko, Xiao Yu Tian, Patrick Ming-Kuen Tang, Kathy O. Lui

**Affiliations:** ^1^Department of Chemical Pathology, Prince of Wales Hospital, The Chinese University of Hong Kong, Hong Kong, China; ^2^State Key Laboratory of Translational Oncology, Department of Anatomical and Cellular Pathology, Prince of Wales Hospital, The Chinese University of Hong Kong, Hong Kong, China; ^3^School of Biomedical Sciences, The Chinese University of Hong Kong, Hong Kong, China; ^4^Department of Medicine and Therapeutics, Prince of Wales Hospital, The Chinese University of Hong Kong, Hong Kong, China; ^5^Li Ka Shing Institute of Health Sciences, Prince of Wales Hospital, The Chinese University of Hong Kong, Hong Kong, China; ^6^Shenzhen Research Institute, The Chinese University of Hong Kong, Shenzhen, China

**Keywords:** agrin, endothelial cell (EC), angiogenesis, tumorigenesis, metastasis

## Abstract

Recently, the extracellular matrix protein agrin has been reported to promote tumor angiogenesis that supports tumorigenesis and metastasis; however, there is a lack of *in vivo* genetic evidence to prove whether agrin derived from the tumors or endothelial cells (ECs) systemically should be the therapeutic target. To date, the physiological role of endothelial agrin has also not been investigated. In the EC-specific agrin knockout mice, we observed normal endothelial and haematopoietic cell development during embryogenesis. Moreover, these mice develop normal vascular barrier integrity and vasoreactivity at the adult stage. Importantly, the growth of localized or metastatic cancer cells was not affected after implantation into endothelial agrin depleted mice. Mechanistically, agrin did not regulate endothelial ERK1/2, YAP or p53 activation *in vivo* that is central to support endothelial proliferation, survival and invasion. Cumulatively, our findings may suggest that agrin could play a redundant role in endothelial development during physiological and tumor angiogenesis. Targeting the endothelial derived agrin might not be effective in inhibiting tumor angiogenesis.

## Introduction

The extracellular matrix (ECM) is a network of secreted macromolecules that forms a non-cellular microenvironment essential in modulating cell behavior during multiple biological processes including development (1), repair and regeneration (2), as well as tumorigenesis and metastasis (3). In endothelial cells (ECs), ECM proteins such as collagens, laminins and heparan sulfate proteoglycans form the basement membrane that not only provides a structural scaffold, but also key signaling events involved in the regulation of endothelial migration, invasion, proliferation and survival [for review, see (4, 5)]. While the biological roles of collagens and laminins are well characterized in ECs, that of surface proteoglycans such as agrin have been less studied.

Agrin is expressed in the basement membrane of multiple organs particularly in the brain, lung and muscle (6). In the central nervous system (CNS), agrin binds to Lrp4 and mediates muscle-specific receptor tyrosine kinase (MuSK) signaling during neuromuscular synapse formation (7, 8). It also regulates acetylcholine receptor clustering required for the function of neuromuscular junction (9). In the cardiovascular system, recent work has demonstrated an essential role of agrin in cardiac regeneration. Although the adult myocardium is notorious in its inability to regenerate, agrin can promote adult cardiomyocyte proliferation and regeneration after myocardial infarction *via* the YAP- and ERK-mediated signaling pathways (10). Besides, it has also been observed that agrin is overexpressed and secreted in tumor cells such as hepatocellular carcinoma (HCC), and is responsible for enhancing tumor cell proliferation and migration *via* the Lrp4/MuSK signaling (11).

Angiogenesis, a process by which new blood vessels are formed by sprouting from the pre-existing ones, requires EC proliferation, invasion and migration. Our previous work shows that the endothelium can regenerate after injury through sprouting angiogenesis (12, 13). In addition, tumor growth and metastasis also depend on angiogenesis that represents a hallmark of cancer. Recently, agrin has been demonstrated to recruit blood vessels within a growing tumor as xenotransplantation of Matrigel plugs containing agrin-depleted human liver cancer cells in immunodeficient mice shows reduced infiltration of murine CD31^+^ ECs compared to that of the control (14). These findings suggest that agrin promotes tumor angiogenesis, and is likely a therapeutic target for cancer therapy. shRNA-mediated agrin knockdown in human umbilical vein ECs (HUVEC) also significantly reduces *in vitro* angiogenesis, proliferation and migration through reduced vascular endothelial growth factor receptor 2 (VEGFR2) expression; and impedes the activation of extracellular signal regulated kinase (ERK)1/2, protein kinase B (Akt), and endothelial nitric oxide synthase (eNOS) signaling downstream of VEGFR2 (14). Nevertheless, these experiments cannot clearly address the role of endothelial agrin in tumor angiogenesis as the recipient's tumor infiltrating ECs are not deficient in agrin. Indeed, agrin is detected in both macro- and microvascular ECs (14), but the role of endothelial agrin in tumor angiogenesis has not been investigated.

In fact, the brain, lung, muscle and tumor where agrin expression is detected are highly vascularised tissues; however, the *in vivo* function of endothelial agrin during development and pathophysiological angiogenesis remains unclear. Herein, we utilized a genetic tool to specifically deplete endothelial agrin in *Cdh5-Cre;Agrn*^*fl*/*fl*^ mice. We report that endothelial specific ablation of agrin does not impair endothelial and haematopoietic cell development during embryogenesis. Moreover, endothelial loss of agrin in adult mice does not weaken vascular integrity nor vasoreactivity *in vivo*; and does not inhibit tumor angiogenesis and metastasis. Collectively, our findings may suggest that agrin is dispensable for endothelial development and angiogenesis during homeostasis and tumorigenesis, respectively.

## Results

### Endothelial Agrin Is Dispensable for Endothelial and Haematopoietic Cell Development

**Figure 1:**
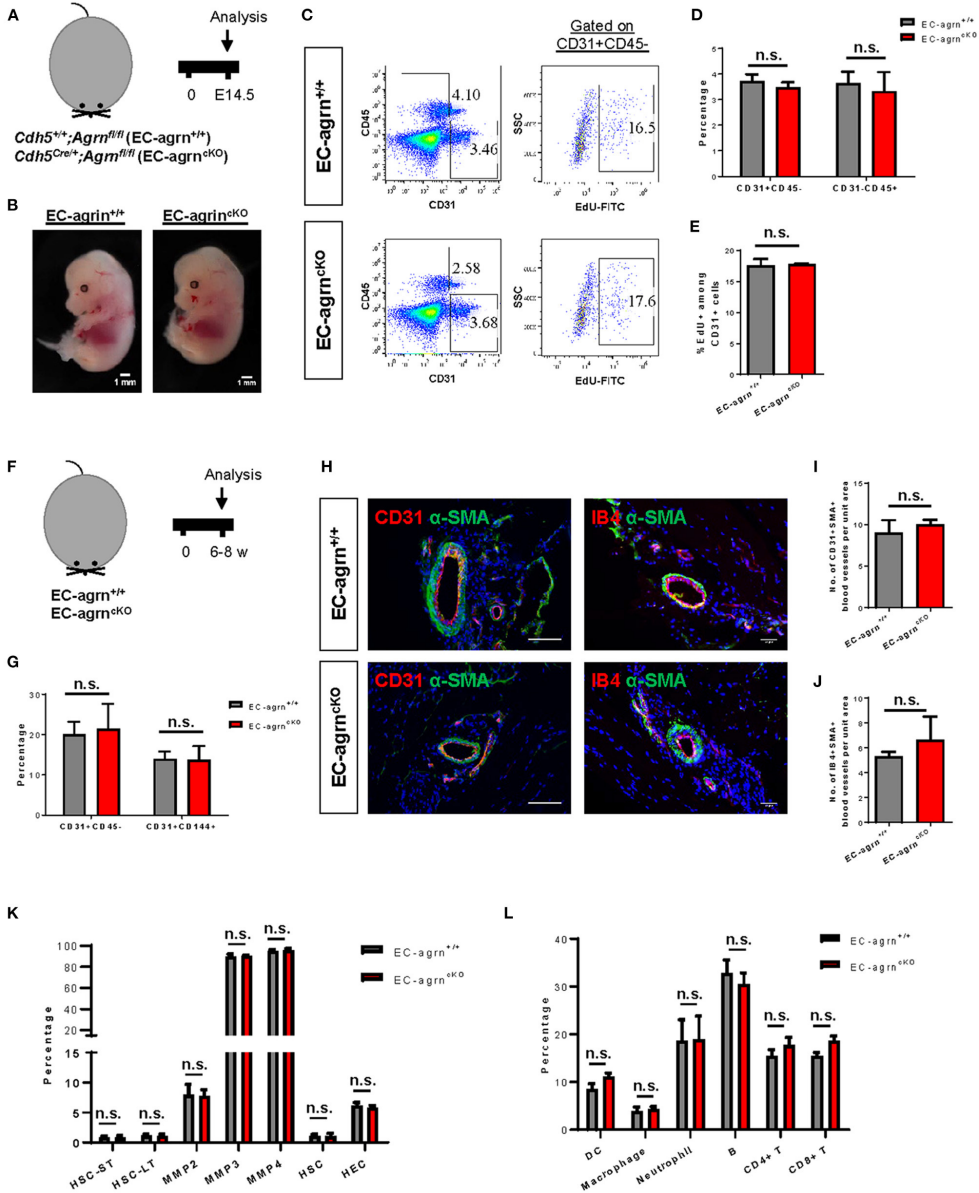
Endothelial agrin is dispensable for endothelial and haematopoietic cell development. **(A)** Schematic diagram showing the experimental design in embryos of *Cdh5*^*Cre*/+^*;Agrn*^*fl*/*fl*^ (EC-agrn^cKO^) and *Cdh5*^+/+^*;Agrn*^*fl*/*fl*^ (control, EC-agrn^+/+^) mice. **(B)** Embryos harvested at embryonic day **(E)** 14.5 showing no gross difference after endothelial loss of agrin, scale bars: 1 mm. **(C)** Flow cytometric analyses showing the distribution of CD31^+^CD45^−^ ECs, CD31^−^CD45^+^ haematopoietic cells, or CD31^+^CD45^−^EdU^+^ proliferating ECs purified from the embryos. **(D,E)** Quantification of **(C)** showing no significant difference in the percentage of **(D)** ECs and haematopoietic cells or **(E)** proliferating ECs after endothelial loss of agrin in embryos. **(F)** Schematic diagram showing experimental design in adult mice. **(G)** Quantification of flow cytometric analyses showing no significant difference in the percentage of ECs and haematopoietic cells after endothelial loss of agrin in adult mice. **(H)** Immunostaining on frozen sections for α-SMA (green), nuclear counterstain by DAPI (blue) and CD31 (red) or IB4 (red), scale bars: 100 um. **(I,J)** Quantification of **(H)** showing no significant difference in the absolute number of **(I)** CD31^+^αSMA^+^ or **(J)** IB4^+^αSMA^+^ blood vessels per unit area after endothelial loss of agrin in adult mice. **(K,L)** Quantification of flow cytometric data showing no significant difference in the percentage of **(K)** various haematopoietic stem or progenitor cells or **(L)** various white blood cell lineages after endothelial loss of agrin in adult mice. **(D,E,G,I–L)** Data are presented as mean ± S.E.M., *n* = 3–4, differences were determined by ANOVA and Turkey's HSD *post-hoc* test, n.s. denotes no significant difference.

To investigate the role of endothelial agrin in EC development during embryogenesis, we crossed *Agrn*^*fl*/*fl*^ with *Cdh5*^*Cre*/+^ to generate the conditional knockout line, *Cdh5*^*Cre*/+^;*Agrn*^*fl*/*fl*^ (*EC-agrn*^*fl*/*fl*^), in which agrin was depleted in ECs compared to the control mice, *Cdh5*^+/+^;*Agrn*^*fl*/*fl*^ (*EC-agrn*^+/+^). We first performed timed mating and harvested the embryos at embryonic day (E) 14.5 (Figure 1A). A gross evaluation of the embryos was performed and no difference in size or appearance was observed in the *EC-agrn*^*fl*/*fl*^ compared to *EC-agrn*^+/+^ embryos (Figure 1B). Since haematopoietic cells are derived from ECs in the process known as endothelial-to-haematopoietic transition (EHT) during embryogenesis (15), we would anticipate reduced EHT and, therefore, less CD45^+^ haematopoietic cells if EC development was impaired after endothelial loss of agrin. Flow cytometric analysis was performed to determine the proportion of ECs and haematopoietic cells at E14.5 (Figure 1C). There was no significant difference in %CD31^+^CD45^−^ ECs nor %CD31^−^CD45^+^ haematopoietic cells of the *EC-agrn*^*fl*/*fl*^ compared to *EC-agrn*^+/+^ embryos (Figure 1D). In addition, we evaluated the rate of EC proliferation by flow cytometry (Figure 1C) and found no significant difference in %CD45^−^CD31^+^EdU^+^ cells of the *EC-agrn*^*fl*/*fl*^ compared to that of the *EC-agrn*^+/+^ ones (Figure 1E).

Previous work has demonstrated that agrin is highly enriched in the skeletal muscle (6). Therefore, we also evaluated EC development in the adult skeletal muscle of 6–8 weeks old mice (Figure 1F). Flow cytometric analysis demonstrated no significant difference in %CD31^+^CD45^−^ nor %CD144^+^CD31^+^ ECs of *EC-agrn*^*fl*/*fl*^ compared to that of *EC-agrn*^+/+^ mice (Figure 1G). Immunostaining for EC-specific marker CD31 or isolectin B4 (IB4) with smooth muscle cell-specific marker α-smooth muscle actin (α-SMA) revealed no significant difference in the number of CD31^+^α-SMA^+^ (Figures 1H,I) nor IB4^+^α-SMA^+^ (Figures 1H,J) blood vessels derived from *EC-agrn*^*fl*/*fl*^ compared to that of *EC-agrn*^+/+^ mice. Furthermore, we also examined the development of haematopoietic progenitor cells and different lineages of the white blood cells in adult mice after endothelial agrin ablation. Flow cytometric analysis respectively revealed no significant difference in short-term and long-term haematopoietic stem cells (HSC-ST/LT), different subsets of multipotent progenitors (MPP), and ckit^+^CD34^+^ haematopoietic stem and progenitor cells (HSPCs) from the bone marrow (Figure 1K); as well as CD11c^+^ dendritic cells (DC), CD11b^+^F4/80^+^ macrophages, CD11b^+^Ly6G^+^ neutrophils, B220^+^CD19^+^ B, CD3^+^CD4^+^ helper T and CD3^+^CD8^+^ cytotoxic T-cells among total CD45^+^ cells in the blood (Figure 1L) of adult *EC-agrn*^*fl*/*fl*^ compared to that of *EC-agrn*^+/+^ mice. Taken together, our results suggested that endothelial agrin was dispensable for embryonic and adult endothelial and haematopoietic development, respectively.

### Endothelial Loss of Agrin Does Not Weaken Vascular Barrier Integrity *in vivo*

**Figure 2:**
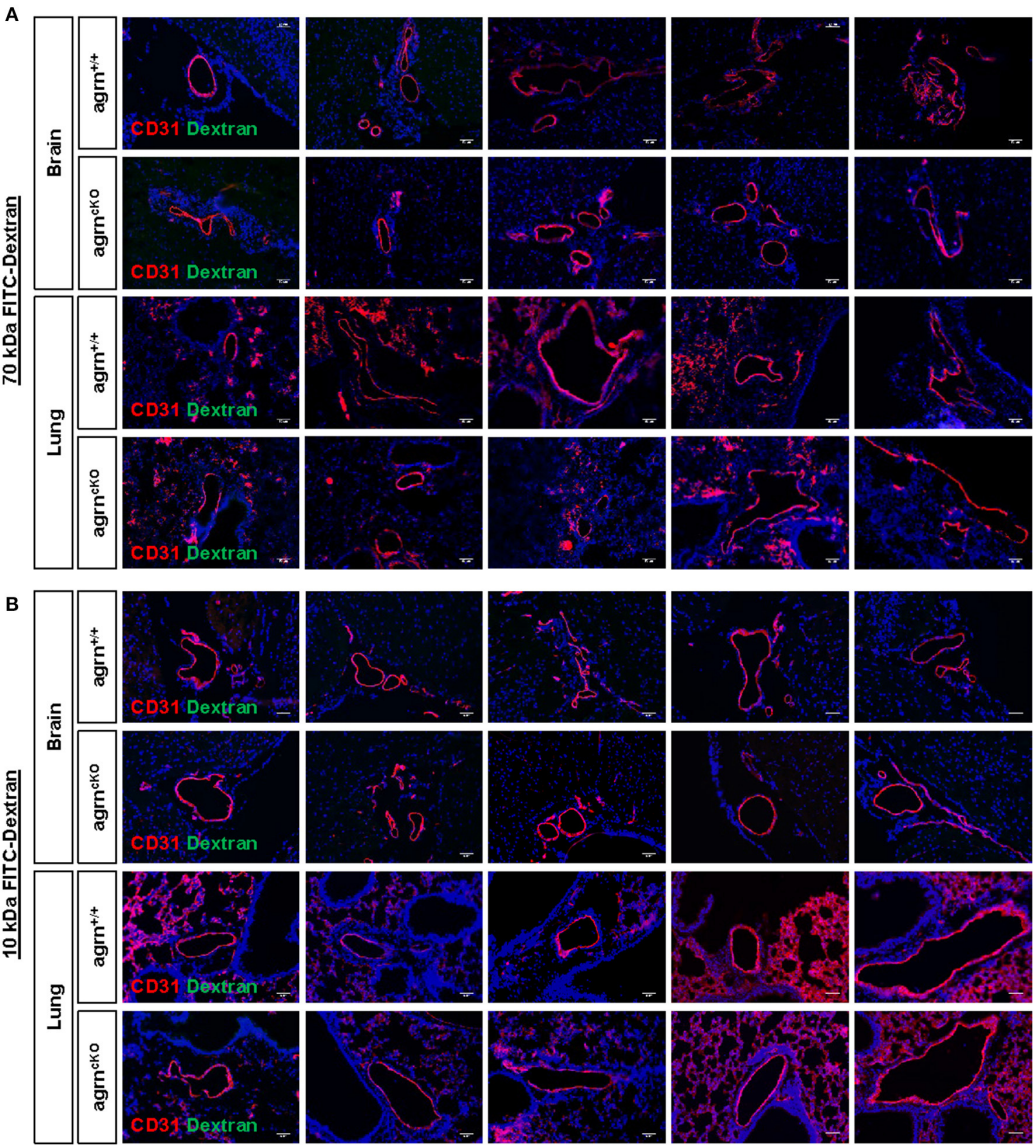
Endothelial agrin is dispensable for the maintenance of vascular barrier integrity *in vivo*. The *in vivo* vascular permeability was examined at 30 min after intravenous injection of **(A)** 70 and **(B)** 10 kDa FITC conjugated Dextran into the tail veins of 6–8 weeks old EC-agrn^+/+^ and EC-agrn^cKO^ mice, respectively. Immunostaining on brain and lung frozen sections for CD31 (red), dextran (green) and nuclear counterstain by DAPI (blue) respectively showing no significant difference in vascular barrier integrity *in vivo* after endothelial loss of agrin, scale bars: 50 um.

Endothelial dysfunction is commonly characterized by increased permeability as a result of the loss of endothelial barrier integrity. Previous work has demonstrated that agrin is highly enriched in the brain and lung tissues (6). To ask if endothelial agrin regulates vascular permeability in the brain and lung tissues, we examined the endothelial junctional alternations at 30 min after intravenous injection of the tracers, 70 and 10 kDa FITC-labeled Dextran, respectively. Significantly increased levels of FITC from the circulation to the surrounding tissues would indicate increased extravasation. Compared to *EC-agrn*^+/+^, there was no leakage of the 70 (Figure 2A) nor 10 (Figure 2B) kDa FITC-Dextran into the brain and lung tissues localized in close proximity to the CD31^+^ endothelium of *EC-agrn*^*fl*/*fl*^, respectively. Moreover, no thinning or obvious gap was observed in the endothelium of *EC-agrn*^*fl*/*fl*^ compared to that of *EC-agrn*^+/+^, suggesting that the loss of endothelial agrin did not compromise vascular barrier integrity. Altogether, our results indicated that agrin might not control endothelial barrier function; or the loss of endothelial agrin was compensated by other mediators to maintain the barrier integrity.

### Endothelial Loss of Agrin Does Not Impair Endothelium-Dependent Vasoreactivity *in vivo*

**Figure 3:**
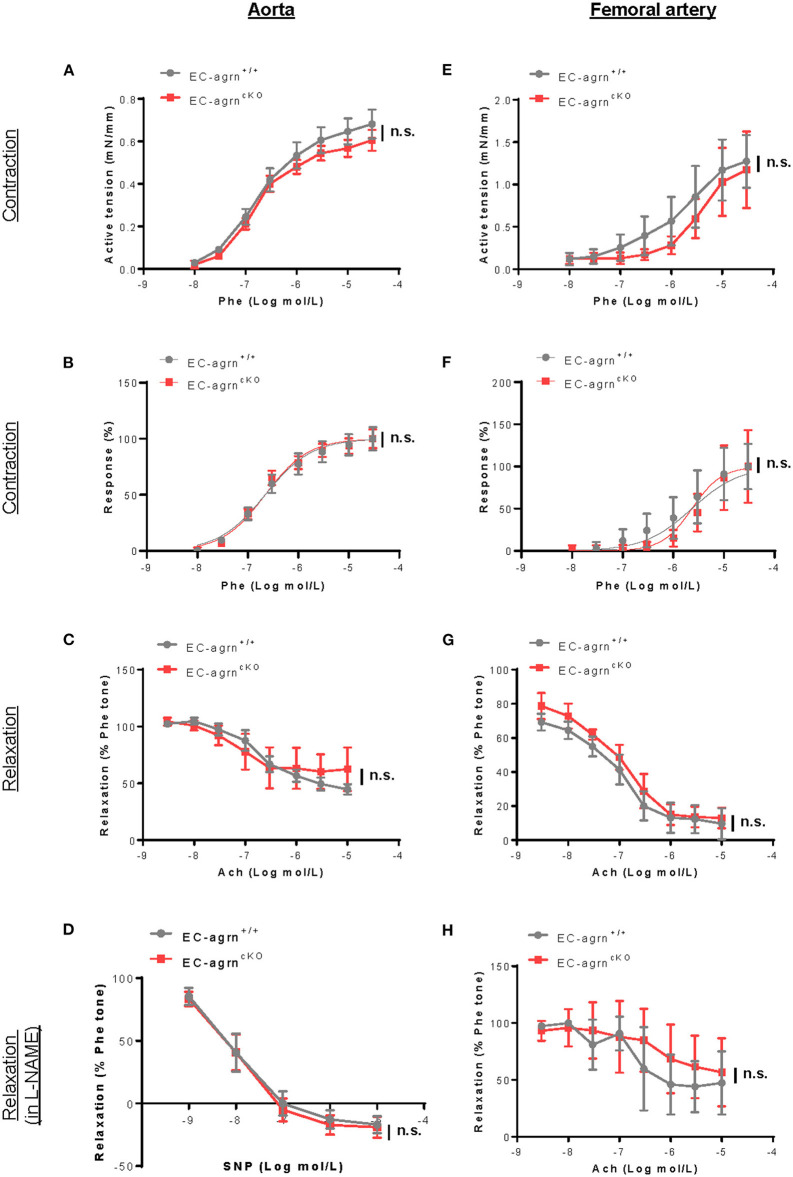
Endothelial agrin is dispensable for endothelium dependent vasoreactivity *in vivo*. **(A–F)** Concentration dependent vasoreactivity measured in the **(A–D)** aorta and **(E–H)** femoral artery of 6-8 weeks old EC-agrn^+/+^ and EC-agrn^cKO^ mice, respectively, showing no significant difference after endothelial loss of agrin. **(A,E)** Concentration dependent vasocontractions of mouse aorta or femoral artery of the limb in response to phenylephrine (Phe) were measured. **(B,F)** Dose response curve showing EC50 of Phe-induced vasocontractions. **(C,G)** Concentration dependent EDRs of mouse aorta or femoral artery of the limb in response to acetylcholine (Ach) were measured. **(D,H)** Reactions were antagonized by co-treatment with NOS inhibitor, L-NAME. **(A–H)** Data are presented as mean ± S.E.M., *n* = 5–6, differences were determined by ANOVA and Turkey's HSD *post-hoc* test, n.s. denotes no significant difference.

Endothelial dysfunction is also characterized by reduced vascular relaxation. To elucidate the role of endothelial agrin in endothelium-dependent vasoreactivity, we respectively performed wire myography in the aorta and femoral artery of 6–8 weeks old mice. There was no significant difference in phenylephrine (Phe)-mediated vasocontractions in terms of active tension (Figure 3A) or dose responsiveness (Figure 3B); or acetylcholine (ACh)-mediated, endothelium-dependent relaxations (EDR, Figure 3C) by the aorta of *EC-agrn*^*fl*/*fl*^ compared to that of *EC-agrn*^+/+^ mice. Moreover, there was no significant difference in sodium nitroprusside (SNP)-mediated EDR after treatment with the nitric oxide synthase (NOS) inhibitor L-NAME that blocks endothelial NO production responsible for vascular relaxations (Figure 3D). Similarly, there was no significant difference in Phe-mediated vasocontractions (Figures 3E,F); ACh-mediated EDR without (Figure 3G) or with L-NAME (Figure 3H) by the femoral artery of *EC-agrn*^*fl*/*fl*^ compared to that of *EC-agrn*^+/+^ mice. Altogether, our findings suggested that endothelial agrin did not regulate vasoreactivity; or the loss of endothelial agrin was compensated by other mediators in the maintenance of endothelium dependent vasoreactivity.

### Endothelial Agrin Is Dispensable for Tumor Angiogenesis During Tumor Growth and Metastasis

**Figure 4:**
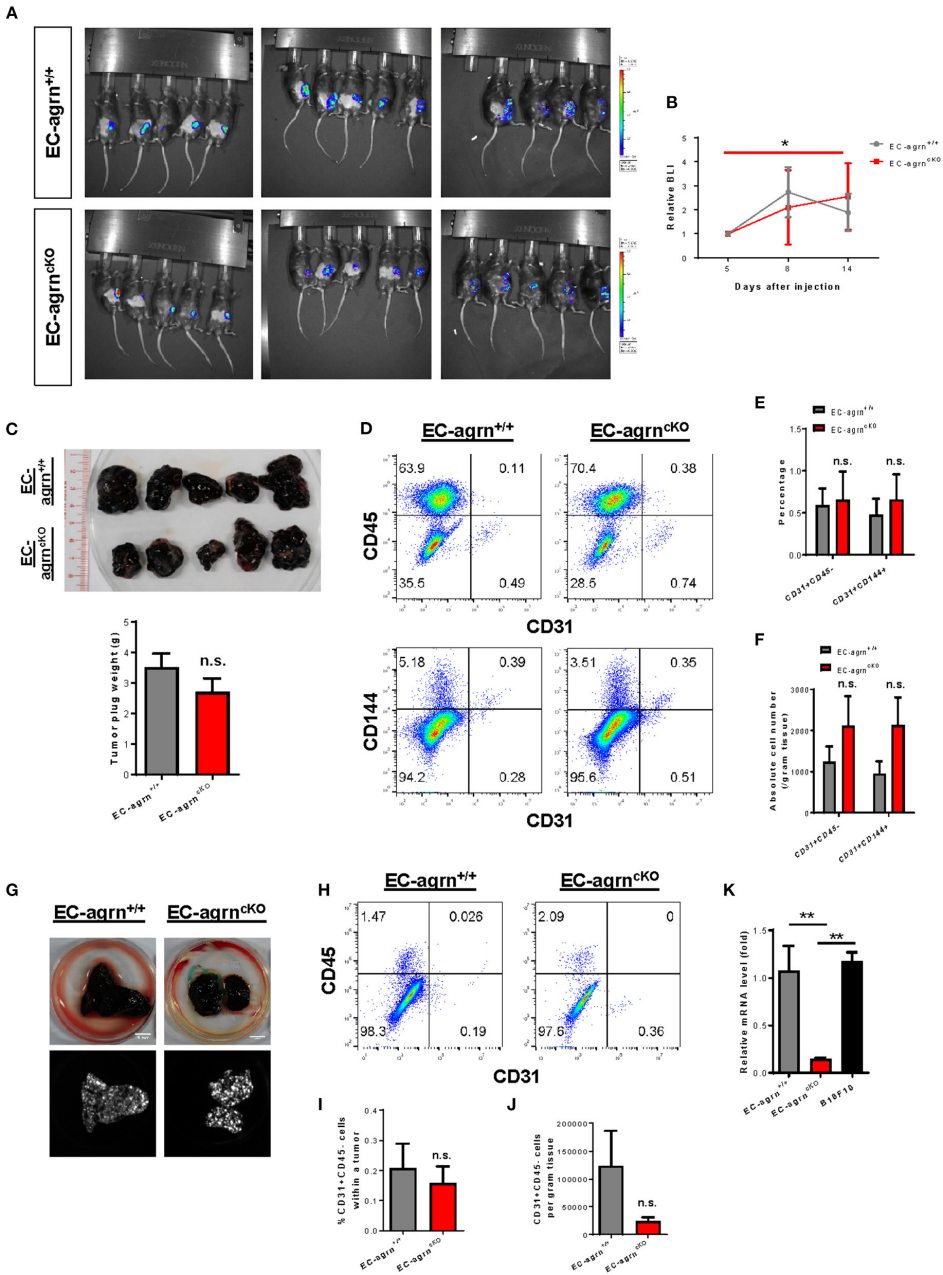
Endothelial agrin is dispensable for tumor angiogenesis during tumor growth and metastasis. **(A)** Longitudinal *in vivo* optical bioluminescence imaging (BLI) showing the survival and growth of *luciferase*-expressing B16F10 melanoma cells (1 x 10^6^ cells per mouse) at day 5, 8 and 14 after subcutaneous implantation into 6–8 weeks old EC-agrn^+/+^ and EC-agrn^cKO^ mice, respectively. **(B)** Quantification of **(A)** at the indicated time points showing no significant difference in localized tumor mass after endothelial loss of agrin. **(C)** The macroscopic morphology and tumor weights were measured on day 14 showing no significant difference after endothelial loss of agrin. **(D)** Flow cytometric analyses showing the distribution of CD31^+^CD45^−^ and CD31^+^CD144^+^ ECs derived from the skeletal muscle of EC-agrn^+/+^ and EC-agrn^cKO^ mice, respectively. **(E,F)** Quantification of **(D)** showing no significant difference in the **(E)** percentage or **(F)** absolute number of CD31^+^CD45^−^ and CD31^+^CD144^+^ ECs per gram tissue after endothelial loss of agrin. **(G)** The macroscopic morphology of the lungs and luciferase signals of the tumors at day 11 after intravenous injection of B16F10 melanoma cells (3 x 10^5^ cells per mouse) showing no significant difference in metastatic growth after endothelial loss of agrin. **(H)** Flow cytometric analyses showing the distribution of CD31^+^CD45^−^ ECs derived from the skeletal muscle of EC-agrn^+/+^ and EC-agrn^cKO^ mice, respectively. **(I,J)** Quantification of **(H)** showing no significant difference in the **(I)** percentage or **(J)** absolute number of CD31^+^CD45^−^ ECs per gram tissue after endothelial loss of agrin. **(K)** RT-qPCR analysis showing significantly loss of *agrn* gene expression in the skeletal muscle ECs isolated from EC-agrn^cKO^ mice; but no significant difference in *agrn* expression in the skeletal muscle ECs of EC-agrn^+/+^ mice and B16F10 cells. **(B,C,E,F,I-K)** Data are presented as mean ± S.E.M., *n* = 5, differences were determined by ANOVA and Turkey's HSD *post-hoc* test, *P < 0.05, **P < 0.01, n.s. denotes no significant difference.

Recently, agrin has been demonstrated to recruit blood vessels within a growing tumor as xenotransplantation of Matrigel plugs containing agrin-depleted human liver cancer cells shows reduced infiltration of murine CD31^+^ ECs in immunodeficient mice compared to that transplanted with agrin-expressing cancer cells (14). Moreover, agrin knockdown in human ECs including umbilical vein ECs (HUVEC), retina microvascular ECs (HREC), dermal microvascular ECs (HDMEC) and aortic ECs (HAEC) also demonstrates significantly reduced *in vitro* angiogenesis as evident by reduced tube formation (14). Therefore, the study has elegantly revealed the potential of agrin as a therapeutic target for dampening tumor angiogenesis and metastasis. Nevertheless, whether tumor or endothelial agrin should be targeted *in vivo* remains unclear. To elucidate whether endothelial agrin plays a role in blood vessel recruitment within localized tumors *in vivo*, we introduced a syngeneic tumor implantation model in which one million *firefly luciferase* (Luc)-expressing B16F10 melanoma cells derived from C57BL/6 mice were mixed with Matrigel and injected subcutaneously into C57BL/6 mice. Tumor growth within the Matrigel plugs was monitored by *in vivo* bioluminescent imaging on day 5, 8 and 14 after implantation (Figure 4A). Our results showed that the tumor volumes of *EC-agrn*^*fl*/*fl*^ and *EC-agrn*^+/+^ mice represented by the bioluminescent photon intensities were not significantly different at day 14 after implantation, but there was a significant tumor growth in *EC-agrn*^*fl*/*fl*^ mice comparing day 5 and 14 after implantation (Figure 4B). Nevertheless, there was no gross difference between both groups in terms of size and weight of the Matrigel plugs containing tumors harvested at day 14 after implantation (Figure 4C). To quantify tumor angiogenesis, the tumors of both groups were enzymatically digested into single cells for flow cytometry (Figure 4D). There was also no significant difference in the percentage (Figure 4E) nor absolute number (Figure 4F) of CD31^+^CD45^−^ and CD31^+^CD144^+^ ECs within localized tumors harvested from both groups, respectively.

To examine whether endothelial agrin is required for blood vessel recruitment within metastasizing tumor foci *in vivo*, we inoculated B16F10-Luc cells through the tail veins and monitored their metastasis progression to the lungs as previously described (16). At day 11 after intravenous injection, noticeable lung lesions were developed in both groups, and no significant difference was observed in metastatic dissemination of tumor cells harvested from the lungs of *EC-agrn*^*fl*/*fl*^ and *EC-agrn*^+/+^ mice (Figure 4G). To quantify endothelial contribution to the metastatic lesions, the tumors harvested from the lungs of both groups were enzymatically digested into single cells for flow cytometry (Figure 4H). There was no significant difference in the percentage (Figure 4I) nor absolute number (Figure 4J) of CD31^+^CD45^−^ ECs within tumors harvested from both groups, respectively, indicating that tumor metastasis to the lungs was not dependent on endothelial agrin expression in pulmonary blood vessels. Furthermore, we also determined the potential impact from tumor cell derived agrin on tumor angiogenesis by quantifying *agrin* gene expression in B10F10-Luc cells compared to that of the ECs isolated from *EC-agrn*^*fl*/*fl*^ and *EC-agrn*^+/+^ mice by quantitative RT-qPCR. Similar to SK-N-SH neuroblastoma cells and MHCC-LM3 liver cancer cells (14), the B10F10 melanoma cells also expressed *agrn* at a similar level compared to that of the skeletal muscle ECs of *EC-agrn*^+/+^ mice but at a significantly lower level than that of the skeletal muscle ECs of *EC-agrn*^*fl*/*fl*^ mice (Figure 4K), suggesting that tumor angiogenesis could be rescued by tumor derived agrin even in endothelial agrin deficient mice. Taken together, these data suggested that endothelial agrin was not required for EC recruitment during the early stages of localized and metastatic tumor growth *in vivo* as agrin derived from the tumor could compensate for the loss of endothelial agrin.

### Agrin Is Not Required for Endothelial ERK1/2-, YAP- Nor p53-Mediated Activation *in vivo*

**Figure 5:**
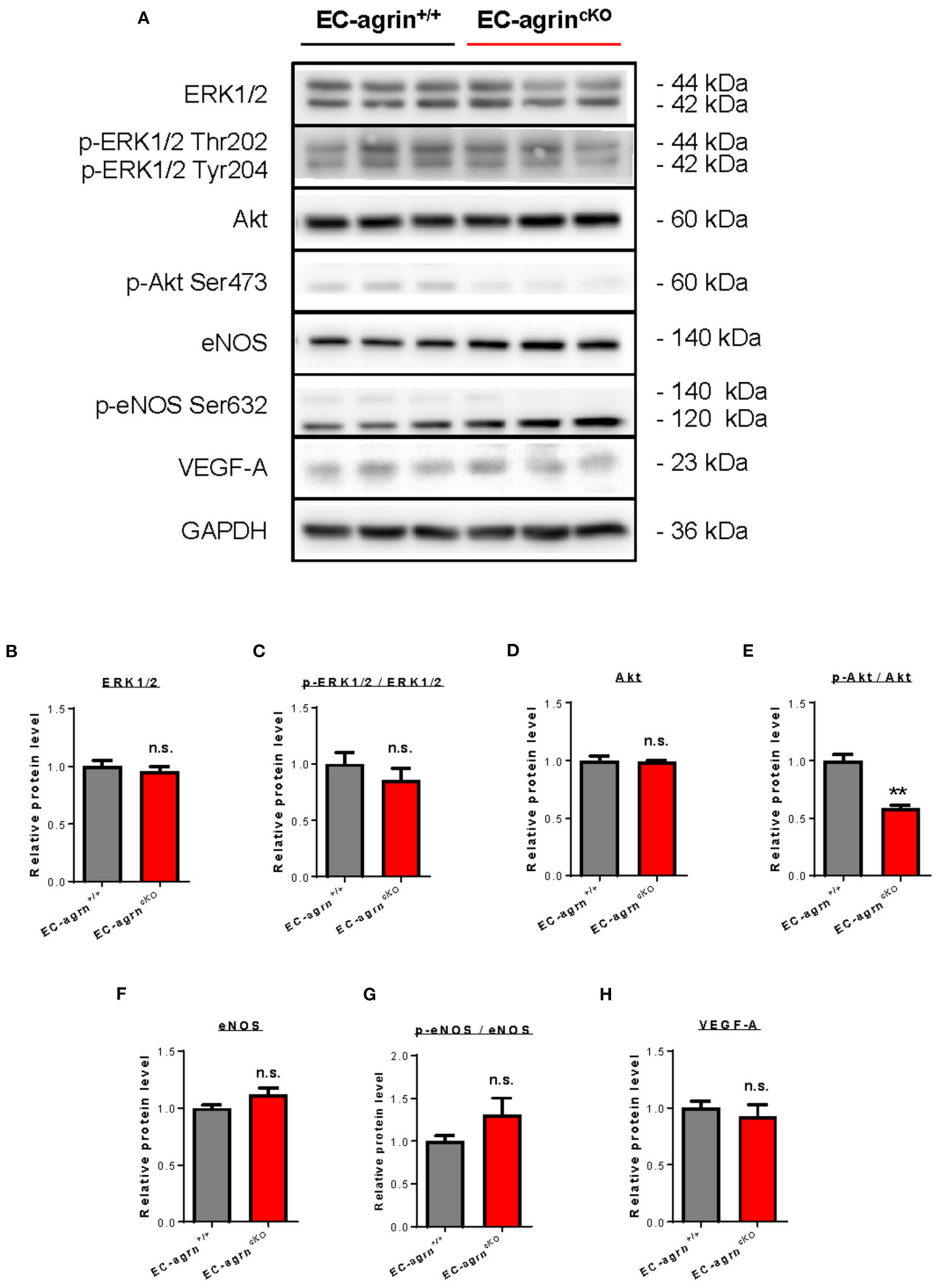
Agrin does not regulate endothelial ERK1/2 mediated activation *in vivo*. **(A)** Western blotting analysis showing no significant difference in endothelial expression and activation of ERK1/2 or eNOS after endothelial loss of agrin *in vivo*. The expression and activation levels of **(B,C)** ERK1/2, **(D,E)** Akt and **(F,G)** eNOS; as well as the expression levels of **(H)** VEGF-A were measured in the skeletal muscle ECs of 6–8 weeks old EC-agrn^+/+^ and EC-agrn^cKO^ mice, respectively. **(B–H)** Data are presented as mean ± S.E.M., *n* = 3, differences were determined by ANOVA and Turkey's HSD *post-hoc* test, **P < 0.01, n.s. denotes no significant difference.

Previous work has showed that agrin promotes cardiomyocyte proliferation through activation of ERK and YAP mediated signaling pathways (10). In endothelial cells, agrin depletion in HUVEC also demonstrates reduced phosphorylation of ERK1/2, Akt and eNOS *in vitro* (14), suggesting a role of endothelial agrin in the activation of these pathways that are central to EC proliferation, invasion and angiogenesis. To validate whether endothelial agrin is essential for ERK1/2 and YAP-mediated activation *in vivo*, we further analyzed the expression and phosphorylation levels of ERK1/2, Akt and eNOS in CD31^+^ ECs purified from the skeletal muscle of *EC-agrn*^*fl*/*fl*^ and *EC-agrn*^+/+^ mice for western blotting, respectively (Figure 5A). The expression levels (Figure 5B) and overall activities (Figure 5C) of ERK1/2 (i.e., p-ERK1/2 at Thr202 and Tyr204/ERK1/2) were not significantly different in both groups. Even though there was a significant reduction in the expression levels (Figure 5D) and overall activities (Figure 5E) of Akt (i.e., p-Akt at Ser473/Akt), the expression levels (Figure 5F) and overall activities (Figure 5G) of eNOS (i.e., p-eNOS at Ser632/eNOS) that is downstream of Akt activation in ECs (17) were not significantly different in ECs of *EC-agrn*^*fl*/*fl*^ compared to that of *EC-agrn*^+/+^ mice. Moreover, there was also no significant difference in the expression levels of the angiogenic factor VEGF-A in both groups (Figure 5H).

**Figure 6:**
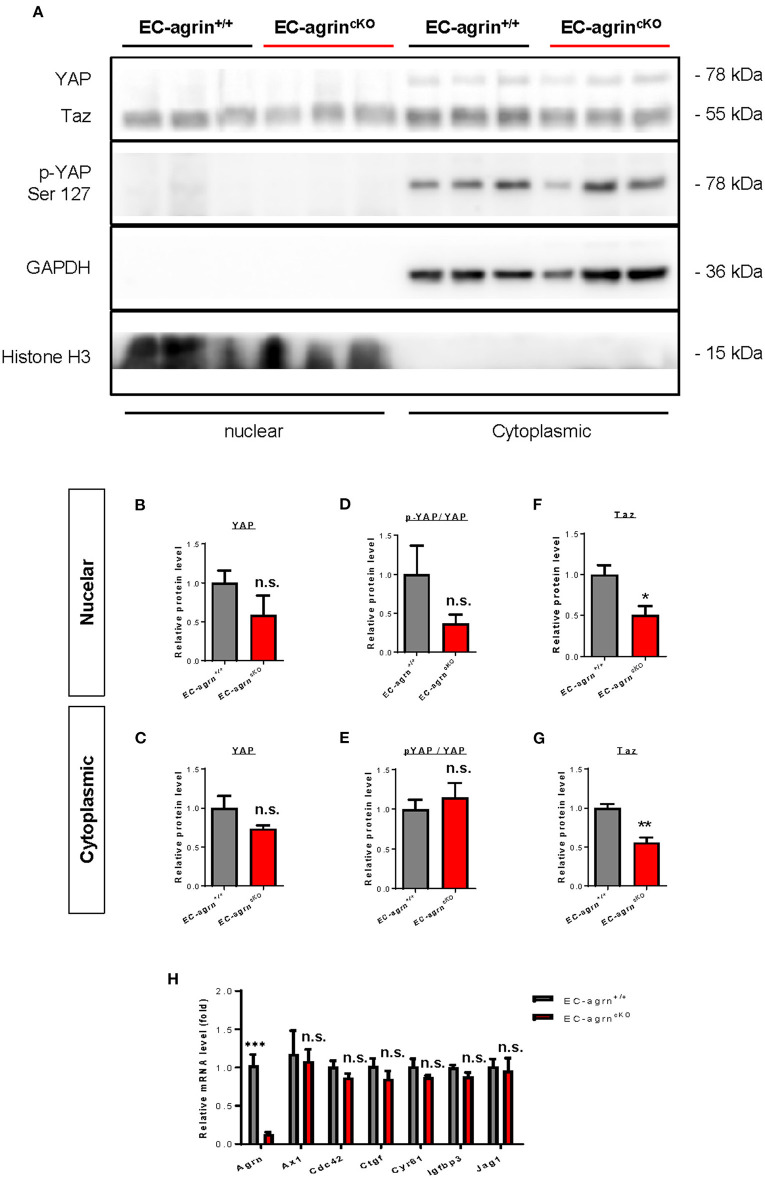
Agrin does not regulate endothelial YAP mediated activation *in vivo*. **(A)** Western blotting analysis showing no significant difference in the nuclear or cytoplasmic distributions of Yap but significantly reduced Taz after endothelial loss of agrin *in vivo*. The nuclear and cytoplasmic **(B,C)** expression and **(D,E)** activation levels of YAP; as well as the **(F)** nuclear and **(G)** cytoplasmic expression levels of Taz were measured in the skeletal muscle ECs of 6–8 weeks old EC-agrn^+/+^ and EC-agrn^cKO^ mice, respectively. **(H)** RT-qPCR analysis showing no significant difference in expression levels of YAP target genes after endothelial loss of agrin. **(B–H)** Data are presented as mean ± S.E.M., *n* = 3-6, differences were determined by ANOVA and Turkey's HSD *post-hoc* test, *P < 0.05, **P < 0.01, ***P < 0.001, n.s. denotes no significant difference.

In addition to the ERK1/2/Akt/eNOS pathways, previous work has also demonstrated that agrin activates YAP/Taz (18). Increased cytoplasmic distribution and enhanced phosphorylation of YAP at Ser127 leading to its proteosomal degradation are observed after agrin knockdown through siRNA or shRNA in liver or breast cancer cells, respectively (18). In ECs, YAP/Taz plays an important role in the regulation of EC development (19), angiogenesis and endothelial barrier maturation (20). Therefore, we sought to understand whether agrin is required for endothelial activation of YAP/Taz *in vivo* by analyzing the expression and phosphorylation levels of nuclear and cytoplasmic YAP and Taz in CD31^+^ ECs purified from the skeletal muscle of *EC-agrn*^*fl*/*fl*^ and *EC-agrn*^+/+^ mice for western blotting, respectively (Figure 6A). The nuclear and cytoplasmic distributions (Figures 6B,C) as well as activation (Figures 6D,E) as evident by p-YAP at Ser127/YAP were not significantly different in ECs of *EC-agrn*^*fl*/*fl*^ and *EC-agrn*^+/+^ mice. On the other hand, both the nuclear (Figure 6F) and cytoplasmic (Figure 6G) distributions of Taz were significantly reduced in ECs of *EC-agrn*^*fl*/*fl*^ than *EC-agrn*^+/+^ mice. Nevertheless, we also examined the expression levels of endogenous YAP target genes downstream of YAP/Taz activation including *Axl* encoding tyrosine protein kinase receptor UFO, cell division cycle 42 (*Cdc42*), connective tissue growth factor (*Ctgf*), cysteine rich angiogenic inducer 61 (*Cyr61*), insulin like growth factor binding protein 3 (*Igfbp3*) and jagged canonical Notch ligand 1 (*Jag1*) as reported previously (18, 21). Whereas the gene expression levels of *Agrn* were significantly reduced in ECs of *EC-agrn*^*fl*/*fl*^ compared to *EC-agrn*^+/+^ mice, there was no significant difference in the expression levels of all these YAP target genes in ECs after depletion of agrin compared to that of the control ECs (Figure 6H).

**Figure 7:**
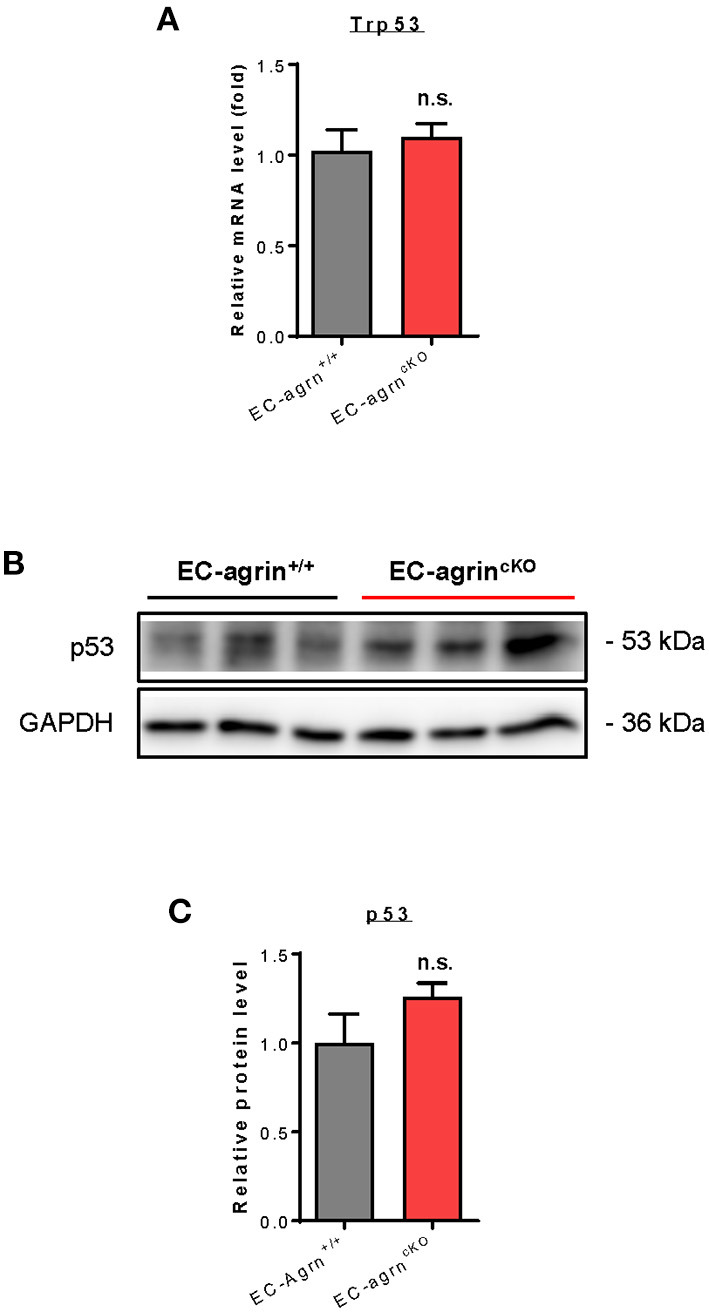
Agrin does not regulate endothelial p53 mediated activation *in vivo*. **(A)** RT-qPCR analysis showing no significant difference in expression levels of Trp53 gene after endothelial loss of agrin *in vivo*. **(B,C)** Western blotting analysis showing no significant difference in endothelial expression of p53 after endothelial loss of agrin *in vivo*. **(A,C)** Data are presented as mean ± S.E.M., *n* = 3, differences were determined by ANOVA and Turkey's HSD *post-hoc* test, n.s. denotes no significant difference.

Recently, it has also been demonstrated that agrin induces scenescent cell accumulation in the epicardium and scar region of regenerating hearts *via* p53 (22). We then examined whether agrin is required for endothelial activation of p53 *in vivo* by analyzing its gene and protein expression levels. The *Trp53* gene (Figure 7A) and p53 protein (Figures 7B,C) expression levels were not significantly different in CD31^+^ ECs purified from the skeletal muscle of *EC-agrn*^*fl*/*fl*^ and *EC-agrn*^+/+^ mice, respectively. Taken together, our findings suggested that endothelial agrin was dispensable for endothelial activation of ERK1/2, YAP or p53 *in vivo*.

## Discussion

In this study, using a genetic tool that enables endothelial specific ablation of agrin, we report that endothelial agrin is not required for EC growth and proliferation, and normal EHT was observed during embryonic development. At the adult stage, endothelial specific loss of agrin did not affect vascular barrier integrity and vasoreactivity. Unlike in other systems such as the heart where agrin is required to promote cardiomyocyte proliferation after injury (10), or in a growing tumor mass where agrin enhances tumor cell proliferation and migration (11, 18), our experiments showed that endothelial agrin was dispensable for proliferation and function of the blood vessel.

Angiogenesis is fundamental to tumor growth and metastasis. During tumor angiogenesis, the initial stages of blood vessel recruitment for vascularizing the expanding tumor requires the proliferation, migration and invasion of ECs (23). Previous reports show that agrin mediates angiogenesis in the tumor microenvironment (14, 24). Transplantation of human cancer cells containing shRNA against agrin expression reduces tumor growth and angiogenesis *in vivo* as evident by reduced numbers of CD31^+^ blood vessel within the growing tumor (14). Moreover, agrin knockdown through siRNA or shRNA in multiple human EC lines including HUVEC, HREC, HDMEC and HAEC demonstrates significantly reduced *in vitro* angiogenesis as demonstrated by reduced tube formation that was rescued by treatment with the soluble agrin (14). Agrin silencing in HUVEC also contributes to reduced endothelial proliferation *in vitro* (14). Collectively, the prior work suggests that agrin could be an essential target for developing cancer therapy through inhibiting EC proliferation and tumor angiogenesis.

Nevertheless, whether the tumor or EC derived agrin should be targeted *in vivo* remains unknown. To address this question, we employed a syngeneic tumor transplantation model in which the Luc expressing melanoma cell line B16F10 was mixed with Matrigel for subcutaneous implantation and plug formation. Unexpectedly, there was no significant difference in the growth of the localized tumor as measured by *in vivo* imaging and in tumor angiogenesis as revealed by intratumor distribution of CD31^+^CD45^−^ ECs using flow cytometry. Furthermore, metastasis of B16F10 cells to the lungs through intravenous injection was not reduced in agrin depleted mice compared to that of the wildtype mice. The amount of CD31^+^CD45^−^ ECs within the metastasized tumor was also not significantly reduced following ablation of agrin in pulmonary blood vessels. Therefore, our findings *in vivo* were not consistent with the previous work that shows reduced *in vitro* angiogenesis following transient knockdown of endothelial agrin in HUVEC (14). We hypothesized that the discrepancy between these *in vitro* and *in vivo* work could be attributed to the availability of agrin from the localized or metastasized tumor that might rescue the loss of agrin in ECs of the *EC-agrn*^*cKO*^ mice during tumor angiogenesis. Indeed, RT-qPCR analysis showed comparable expression levels of *Agrn* in B16F10 cells and ECs isolated from the wildtype *EC-agrn*^+/+^ mice. Collectively, our data indicated that endothelial agrin was not required for EC recruitment during the early stages of localized and metastatic tumor growth *in vivo* as the endothelial loss of agrin could be compensated by the tumor-derived agrin.

**Figure 8:**
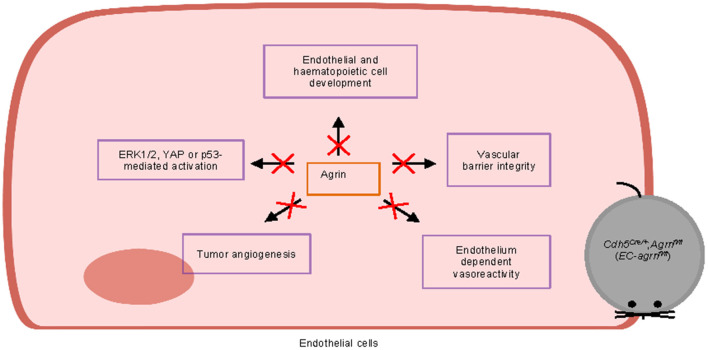
Graphical summary of this study. Endothelial agrin is dispensable for endothelial and haematopoietic cell development, vascular barrier integrity, endothelium dependent vasoreactivity, tumor angiogenesis, and ERK1/2, YAP or p53-mediated activation *in vivo*.

Furthermore, we also examined whether agrin regulates cell signaling in ECs. In contrast to previous reports based on *in vitro* experiments, we found that agrin did not regulate endothelial ERK1/2, YAP or p53 activation *in vivo* that is central to endothelial proliferation and invasion. Taken together, our findings may suggest that agrin could play a redundant role in endothelial development, physiological and tumor angiogenesis (Figure 8). Targeting endothelial agrin might not be effective in inhibiting tumor angiogenesis.

## Experimental Procedures

### Generation of Knockout Mice

The *Agrn*^*fl*/*fl*^ mice described previously (10) were a gift from Robert W. Burgess (The Jackson Laboratory, Bar Harbor, M.E., U.S.A.). To test the effect of agrin in EC development and function, we crossed *Agrn*^*fl*/*fl*^ to *Cdh5*^*Cre*/+^ described previously (12). *Cdh5-Cre;Agrn*^*fl*/*fl*^ mice were maintained on a C57BL/6 background. At the end of experiments, mice were euthanized by cervical dislocation. All animal procedures were approved by the CUHK Animal Experimentation Ethics Committee and performed in accordance with institutional guidelines.

### Cancer Cell Culture

The melanoma B16F10 cell line (ATCC, CRL-6475^TM^) derived from C57BL/6J mice was maintained in DMEM/F12 supplemented with 10% heat inactivated FBS, 1% penicillin and streptomycin in 5% CO_2_ at 37°C. The cells were transduced with a pRRL-CMV-luciferase expressing lentiviral vector as described previously (16).

### *In vivo* Vascular Permeability Assay

Vascular integrity was measured by intravenous injection of 100 μL 40 mg/mL 70 kDa (Sigma, Cat. No. FD70S) or 10 kDa (Sigma, Cat. No. FD10S). FITC-labeled dextran *via* the tail vein of *Cdh5-Cre;Agrn*^*fl*/*fl*^ mice 30 min before sacrifice. Microscopic visualization of dextran extravasation was performed on OCT-embedded tissue sections and co-stained with an anti-CD31 antibody (Biolegend, Cat. No. 102501).

### *In vivo* Vascular Function by Wire Myograph

Vasoreactivity was measured in wire myograph as described previously (12, 13). The aorta or femoral arteries of *Cdh5-Cre;Agrn*^*fl*/*fl*^ mice were removed and dissected in oxygenated ice-cold Krebs solution that contained (in mmol/L): 119 NaCl, 4.7 KCl, 2.5 CaCl_2_, 1 MgCl_2_, 25 NaHCO_3_, 1.2 KH_2_PO_4_, and 11 _D_-glucose. Measurements of isometric tension were recorded in wire myograph (Danish Myo Technology). The arterial segments of aorta and femoral artery were respectively stretched to optimal baseline tension at 3 and 1 mN. After that, they were washed in Krebs solution three times and allowed to equilibrate for 1 h before being contracted with 60 mmol/L KCl to test *via*bility. Endothelium-dependent relaxation (EDR) was measured by testing concentration responses to the cumulative addition of acetylcholine (ACh) in phenylephrine (Phe, 3 μmol/L) pre-contracted segments. Some arteries were incubated with NO synthase inhibitor L-NAME (100 μmol/L, 30 min) before testing vasodilation. Vasoconstriction induced by cumulative concentration of Phe was tested before and after L-NAME, which reflects basal NO production.

### *In vivo* Tumor Angiogenesis Assay

Mice were anesthetized using 2% isoflurane in 100% Oz at 0.5–1 L/min. Cell-free or cell-loaded Matrigel plugs for measuring physiological and tumor angiogenesis, respectively, were prepared as described previously (25–27). For tumor cell-loaded Matrigel plug, 1 million luciferase-expressing B16-F10 cells were suspended in 500 μl Matrigel (Corning Life Sciences, Corning, NY, USA). After that, the mixture was administered subcutaneously to form a syngeneic tumor in *Cdh5-Cre;Agrn*^*fl*/*fl*^ mice. At the end of implantation, single cells were retrieved by digesting the Matrigel plug grafts with a digestion buffer containing collagenase II (11 U/ml; Worthington Biochemical, Lakewood, NJ, USA), dispase (1,000 U/ml; Thermo Fisher Scientific), and DNase I (10 U) at 37°C for 20–30 min. Enzymatic action was stopped by adding 10% FBS, and the dissociated cells were washed twice with phosphate-buffered saline (PBS). The dissociated cells were removed from the contaminated erythrocytes by incubating with red blood cell lysis buffer (eBioscience, San Diego, CA, USA) for 5 min before further experimentations.

### Syngeneic Tumor Metastasis Assay

The syngeneic tumor metastasis model was described previously (16). Briefly, 3 x 10^5^ luciferase-expressing B16-F10 cells were administered intravenously into *Cdh5-Cre;Agrn*^*fl*/*fl*^ or control mice. Mice were observed daily for weight loss, distress, food intake and activities after tumor cell implantation. At the end of implantation, macroscopic morphology was analyzed and luciferase expression was visualized by bioluminescence imaging through the ChemiDoc Imaging System (Biorad).

### Bioluminescence Imaging

Bioluminescence imaging on live mice was previously described (25, 28). Briefly, mice were anesthetized under 2% isoflurane in 100% Oz at 0.5–1 L/min., followed by intraperitoneal injection of 300 mg/kg d-luciferin (Biosynth, St. Gallen, Switzerland). Anesthetized mice were placed in a light-tight chamber of the *In vivo* Xtreme Imaging System (Bruker, Billerica, MA, USA), and photons emitted from live luciferase-expressing cells were captured by the back-illuminated 4 MP camera within 30 min after luciferin injection. Bioluminescent imaging (BLI) signals were expressed in photons/second by drawing a defined region of interest at the site of transplant throughout the experiment with the Bruker MI SE software.

### Flow Cytometry

Embryos were minced into small fragments and dissociated with a digestion buffer containing type II collagenase (500 U/ml, Worthington) and dispase (5.5 U/ml, Gibco) at 37°C for 30 min. Enzymatic action was stopped by adding 10% FBS and the dissociated cells were washed twice with PBS. The dissociated single cells were removed from the contaminated erythrocytes by incubating with the red blood cell lysis buffer (eBiosciences) for 5 min; and were then blocked with 2% normal rabbit serum. Cells were subsequently stained with fluorochrome-conjugated antibodies against the following antigens: B220 (Biolegend, Cat. No. 103211), CD3 (Biolegend, Cat. No. 100204), CD4 (Biolegend, Cat. No. 116012), CD8 (Biolegend, Cat. No. 100712), CD11b (Biolegend, Cat. No. 101207), CD11c (Biolegend, Cat. No. 117309), CD19 (Biolegend, Cat. No. 152407), CD31 (Biolegend, Cat. No. 102508), CD34 (Biolegend, Cat. No. 128611), CD45 (Biolegend, Cat. No. 103106 and 103108), CD48 (Biolegend, Cat. No. 103411), CD144 (Biolegend, Cat. No. 138011), CD150 (Biolegend, Cat. No. 115903), c-Kit (Biolegend, Cat. No. 743384), F4/80 (Biolegend, Cat. No. 123116) or Ly6C (Biolegend, Cat. No. 127613) at a dilution of 1:100, unless specified by the manufacturer, at 4°C for 30 min. For detecting cell proliferation, Alexa Fluor^TM^ 488-labeled EdU (Invitrogen, Cat. No. C10632) was used per manufacturer's instruction. After that, cells were washed three times with 2% FBS-containing PBS and analyzed on flow cytometer (CytoFlex, Beckman Coulter). Propidium iodide (PI, BD) positive dead cells were excluded for live cell analysis/sorting; and FACS data were then analyzed with the FlowJo software (Tree star).

### Immunostaining

The fixed tissues were washed three times with PBS and equilibrated in 30% sucrose at 4°C for 2 days before freezing and cryosectioning. Eight micrometer frozen sections were prepared; and were blocked with 5% BSA and 5% goat serum. After that, the sections were stained with the respective primary antibodies at 10 μg/ml at 4°C overnight. Anti-mouse primary antibodies used: CD31 (Biolegend, Cat. No. 102501), isolectin B4 (Vector Laboratories, Cat. No. FL-1201-0.5) or α-SMA (Dako, Cat. No. M085101-2). Alexa-Fluor-488- or Alexa-Fluor-546-conjugated secondary antibodies (Invitrogen) were used at room temperature for 30 min in the dark. Slides were mounted with DAPI-containing fluorescence mounting medium (Dako) and fluorescence was detected with an upright fluorescence microscope, inverted fluorescence microscope or confocal microscope (all Leica). Images were processed with the ImageJ software.

### Primary EC Isolation

For lung EC isolation, lung tissues were processed as described previously (13). Briefly, lung tissues were removed aseptically, rinsed in PBS, minced into ~1 x 2 mm^2^, and digested in 400 U/ml collagenase II and 5.5 U/ml dispase at 37°C for 45 min with agitation. After that, the suspension was washed twice in EC growth medium (EGM-2, Lonza) and the cell pellet was resuspended and seeded into T25 flask for differential plating. After 1 h of incubation, lung ECs were harvested by removing the supernatant containing non-ECs. For skeletal muscle EC isolation, single fibers were isolated as described previously (12, 13). Skeletal muscles of the femur were minced and enzymatically digested in F10 medium (Sigma) containing 800 U/ml collagenase II (Worthington) and 1% Pen/Strep (Gibco) at 37°C for 1.5 h with agitation. Muscle cells were washed with 10% horse serum (Gibco) and 1% Pen/Strep (Gibco) in F10 medium; and further digested with 11 U/ml dispase (Gibco) and 1,000 U/ml collagenase II at 37°C for 0.5 h with agitation. After that, CD31^+^ skeletal muscle ECs were purified using the CD31 microbeads (Miltenyi Biotec, 130-097-418) per manufacturer's instruction.

### Cell Fractionation

The skeletal muscle ECs were purified by CD31 microbeads followed by incubation on ice for 10 min in a buffer containing 50 mM HEPES-KOH (Sigma, Cat. NO. H0887), 10 mM KCl (Sigma, Cat. NO. P9541), 350 mM sucrose (Sigma, Cat. NO. S8501) and 1 mM EDTA (AMRESCO, Cat. No. 97063-730-EA) with 0.1% Triton X-100, 1 mM dithiothretol (DTT, Vivantis Technologies, Cat. No. PC-0705) and a protease inhibitor cocktail tablet (Sigma, Cat. No. 5892970001) during which the mixture was pipetted up and down in every 3 min. The mixture was then subjected for centrifugation in 2,000 g at 4 °C for 5 min. The supernatant was collected as the cytoplasmic fraction and the pellet was washed with 200 ul buffer twice followed by centrifugation in 2,000 g at 4 °C for 5 min. The pellet was then resuspended in radioimmunoprecipitation assay (RIPA) buffer containing protease inhibitor and 0.5 mM DTT and used as the nuclear fraction for subsequent western blotting.

### Western Blotting

The skeletal muscle ECs were purified at the indicated time points. Protein was extracted using RIPA buffer during a 5-min pipette up-and-down period. Fifteen micrograms of protein were used for analysis. The following dilutions were used for each antibody: Akt (Cell Signaling Technology, Cat. No. 4691T, 1:1000), p-Akt at Ser473 (Cell Signaling Technology, Cat. No. 4060T, 1:1000), ERK1/2 (Cell Signaling Technology, Cat. No. 4965S, 1:1000), p-ERK1/2 at Thr202/Tyr204 (Cell Signaling Technology, Cat. No. 4370S, 1:1000), eNOS (Abcam, Cat. No. ab76198, 1:500), p-eNOS at Ser632 (Abcam, Cat. No. ab76199, 1:500), YAP/Taz (Cell Signaling Technology, Cat. No. 8418S, 1:1000), p-YAP/Taz at Ser127 (Cell Signaling Technology, Cat. No. 13008T, 1:1000), p53 (Cell Signaling Technology, Cat. No. 2524S, 1:1000), GAPDH (Sigma, Cat. No. G9545, 1:5000) or b-actin (ImmunoWay, Cat. No. YM3028, 1:7000). The relative band intensities were quantified using the ImageJ software.

### RNA Isolation and Real Time RT-qPCR

Total mRNA was isolated from the purified lung or skeletal muscle ECs using the RNeasy Mini Kit (QIAGEN) and reverse transcribed using the iScript cDNA Synthesis Kit (Bio-Rad), according to the manufacturer's instructions. Real time reverse transcriptase qPCR (RT-qPCR) was analyzed on the CFX Connect Read-Time PCR Detection system (Bio-Rad) *via* SYBR Green (Bio-Rad). Gene expression levels were normalized to the housekeeping gene *Gapdh* or β*-actin*. The relative gene expression level of each sample was compared with an internal control. Primers used are listed in Supplementary Table 1.

### Statistical Analysis

The data were expressed as arithmetic mean±S.E.M. of biological replicates (*n* = 5, unless otherwise specified) performed under the same conditions. Statistical analysis was performed using the unpaired student's *t*-test with data from two groups; while date from more than two groups was performed using an ANOVA followed by Tukey's method for multiple comparisons. Significance was accepted when *P* < 0.05.

## Data Availability Statement

The raw data supporting the conclusions of this article will be made available by the authors, without undue reservation.

## Ethics Statement

All animal procedures were reviewed and approved by the CUHK Animal Experimentation Ethics Committee and performed in accordance with institutional guidelines.

## Author Contributions

PY, ZF, JY-C, and XC performed experiments. HK, XT, and PT contributed reagents. PY and KL designed experiments, analyzed, and interpreted the data. KL supervised the research and wrote the manuscript. All authors contributed to the article and approved the submitted version.

## Funding

This work was supported by National Natural Science Foundation of China (81922077 and 82070494), Research Grants Council of Hong Kong (14100021, 14108420, C4026-17WF, and M-402-20), University Grants Committee Research Matching Grant Scheme (2019, 2020, and 2021), and Croucher Foundation Innovation Award; Faculty Innovation Award, Young Researcher Award, Research Committee Funding, Direct Grants, and postgraduate studentships (PY, ZF) from CUHK.

## Conflict of Interest

The authors declare that the research was conducted in the absence of any commercial or financial relationships that could be construed as a potential conflict of interest. The handling editor declared a past co-authorship with the authors KL and XT.

## Publisher's Note

All claims expressed in this article are solely those of the authors and do not necessarily represent those of their affiliated organizations, or those of the publisher, the editors and the reviewers. Any product that may be evaluated in this article, or claim that may be made by its manufacturer, is not guaranteed or endorsed by the publisher.
