# Supplementary material for: Endothelial Agrin Is Dispensable for Normal and Tumor Angiogenesis
Source: Front Cardiovasc Med. 2022 Jan 31;8:810477. doi: 10.3389/fcvm.2021.810477 (PMC8841877; doi:10.3389/fcvm.2021.810477)
Supplement: Supplementary file 1 [file Data_Sheet_1.PDF]

Table S1 Primer sequences used in this study

Gene name	Forward	Reverse
Agrn	5’GGACTCAGAAGGCTCCAACTGT3’	5’GTCAGCCATCTCTGGTGTGTAG3’
Axl	5’GGTGTTTGAGCCAACCGTGGAA3’	5’GCCACCTTATGCCGATCTACCA3’
Cdc42	5’GATTGGTGGAGAGCCATACACTC3’	5’TGAGGATGGAGAGACCACTGAG3’
Ctgf	5’TGCGAAGCTGACCTGGAGGAAA3’	5’CCGCAGAACTTAGCCCTGTATG3’
Cyr61	5’GTGAAGTGCGTCCTTGTGGACA3’	5’CTTGACACTGGAGCATCCTGCA3’
Igfbp3	5’CCTCAATGTGCTGAGTCCCAGA3’	5’CTTGTCCACACACCAGCAGAAG3’
Jag1	5’TGCGTGGTCAATGGAGACTCCT3’	5’TCGCACCGATACCAGTTGTCTC3’
